# *N*,*N*-Dicyclo­hexyl-4-nitro­benzamide

**DOI:** 10.1107/S1600536812035660

**Published:** 2012-08-25

**Authors:** Sohail Saeed, Naghmana Rashid, Ray J. Butcher, Sema Öztürk Yildirim, Rizwan Hussain

**Affiliations:** a Department of Chemistry, Research Complex, Allama Iqbal Open University, Islamabad 44000, Pakistan; b Department of Chemistry, Howard University, 525 College Street NW, Washington DC 20059, USA; c Department of Physics, Faculty of Sciences, Erciyes University, 38039 Kayseri, Turkey; d National Engineering & Scientific Commission, PO Box 2801, Islamabad, Pakistan

## Abstract

The title compound, C_19_H_26_N_2_O_3_, crystallizes with two independent mol­ecules in the asymmetric unit which differ in the twist of the phenyl rings with respect to the plane of the amide group [the C—C—C—O torsion angles are 121.5 (3) and −119.6 (3)° in the two mol­ecules. Both cyclo­hexane rings adopt chair conformations. In the crystal, weak C—H⋯O inter­actions occur. The crystal studied was a non-merohedral twin with a minor component of 4.8 (1)%.

## Related literature

For background to *N*-substituted benzamides, see Priya *et al.* (2005 ▶). For conformational analysis, see: Cremer & Pople, (1975 ▶). For related structures, see: Toda *et al.* (1987 ▶); Saeed *et al.* (2011 ▶).
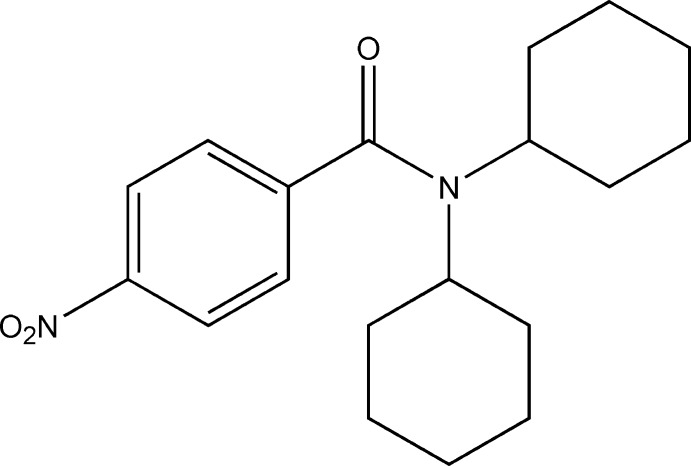


## Experimental

### 

#### Crystal data

C_19_H_26_N_2_O_3_
*M* *_r_* = 330.42
Triclinic, 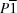

*a* = 6.1874 (3) Å
*b* = 10.7109 (4) Å
*c* = 26.8188 (11) Å
α = 79.128 (4)°
β = 89.027 (4)°
γ = 82.883 (3)°
*V* = 1731.97 (13) Å^3^
*Z* = 4
Cu *K*α radiation
μ = 0.69 mm^−1^
*T* = 123 K
0.29 × 0.26 × 0.07 mm

#### Data collection

Agilent Xcalibur Ruby Gemini diffractometer
Absorption correction: analytical (Clark & Reid, 1995 ▶) *T*_min_ = 0.823, *T*_max_ = 0.950
12453 measured reflections
6952 independent reflections
6374 reflections with *I* > 2σ(*I*)
*R*_int_ = 0.028

#### Refinement

*R*[*F* ^2^ > 2σ(*F* ^2^)] = 0.080
*wR*(*F* ^2^) = 0.239
*S* = 1.11
6952 reflections
434 parameters
H-atom parameters constrained
Δρ_max_ = 0.41 e Å^−3^
Δρ_min_ = −0.29 e Å^−3^

### 

Data collection: *CrysAlis PRO* (Agilent, 2011 ▶); cell refinement: *CrysAlis PRO*; data reduction: *CrysAlis PRO*; program(s) used to solve structure: *SHELXS97* (Sheldrick, 2008 ▶); program(s) used to refine structure: *SHELXL97* (Sheldrick, 2008 ▶); molecular graphics: *SHELXTL* (Sheldrick, 2008 ▶); software used to prepare material for publication: *SHELXTL*.

## Supplementary Material

Crystal structure: contains datablock(s) I, global. DOI: 10.1107/S1600536812035660/ng5286sup1.cif

Structure factors: contains datablock(s) I. DOI: 10.1107/S1600536812035660/ng5286Isup2.hkl

Supplementary material file. DOI: 10.1107/S1600536812035660/ng5286Isup3.cml

Additional supplementary materials:  crystallographic information; 3D view; checkCIF report

## Figures and Tables

**Table 1:** Hydrogen-bond geometry (Å, °)

*D*—H⋯*A*	*D*—H	H⋯*A*	*D*⋯*A*	*D*—H⋯*A*
C5*B*—H5*BA*⋯O3*B* ^i^	0.95	2.54	3.148 (4)	122

Symmetry code: (i) 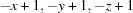.

**Fig. 1.**
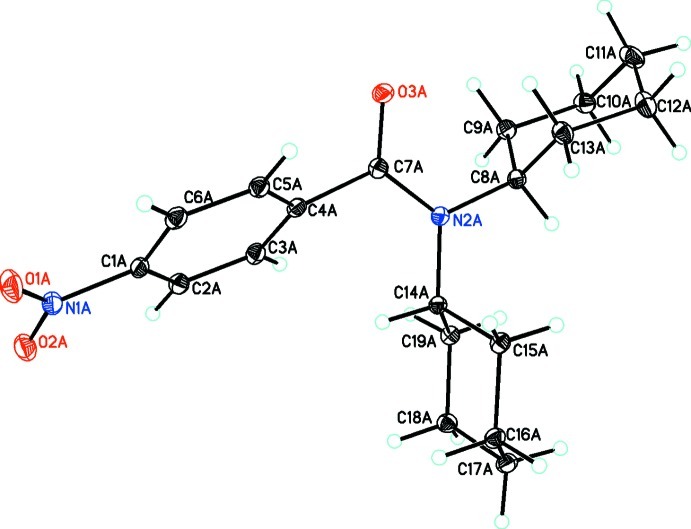
Molecular structure of molecule A in (I) showing the atom labeling scheme and 30% probability displacement ellipsoids.

**Fig. 2.**
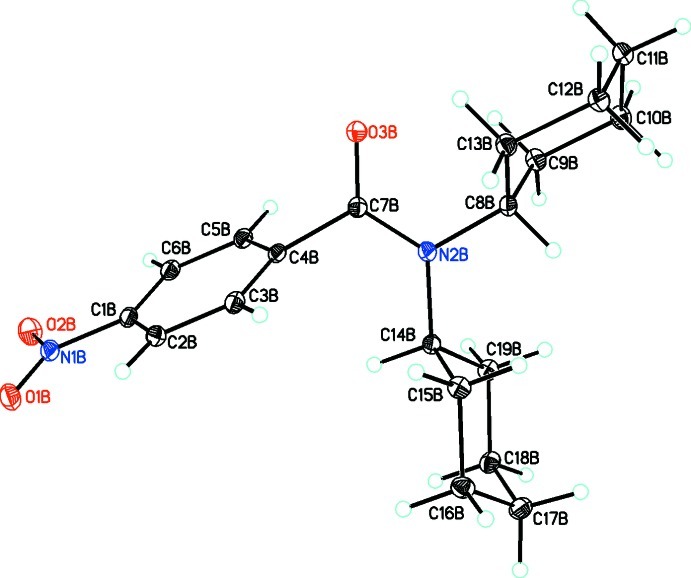
Molecular structure of molecule B in (I) showing the atom labeling scheme and 30% probability displacement ellipsoids.

## supplementary crystallographic information

### Comment

In (I), C_19_H_26_N_2_O_3_, there are two independent molecules (A and B) in the asymmetric unit of the title compound. In molecule A and B the nitro group is almost coplanar with the attached benzene ring in both molecules [C1—C6—N1—O1 = 1.2 (4) ° and C1—C2—N1—O2 = 3.9 (4) ° in molecule A, -3.5 (4) ° and -0.7 (4) ° in molecule B, respectively]. In each molecule, the cyclohexyl rings both adopt a chair conformation as indicated by the puckering parameters Q(2) and φ(2) (Cremer & Pople, 1975) which are 0.012 (1) Å and 177.369 (1) ° in C8A—C13A, 0.015 (1) Å and 122.495 (1) ° in C14A—C19A; 0.017 (1) Å, 21.210 (1) ° in C8B—C13B and 0.013 (1) Å, 230.094 (1) ° in C14A—C19A, respectively. In both molecules, the nitrobenzamide moiety (O3, N2, C7, C8 and C14) is planar (maximum deviation for N2, 0.049 (2) and 0.047 (2) Å in molecules A and B respectively). The dihedral angles between these planar nitrobenzamide moieties and the nitrophenyl groups in molecules A and B are 58.61 (11) and 59.95 (12)° respectively. The main difference in the two molecules lies in how the nitrophenyl rings are arranged with respect to the rest of the molecule and is shown in Figures 1 & 2. This is best illustrated by considering the C3 C4 C7 O3 torsion angle which is 121.46 (33)° in molecule A and -119.62 (34)° in molecule B, thus the phenyl groups in each molecule are twisted in different directions with respect to the plane of the amide moiety. Apart from this the bond lengths and angles in the two molecules agree within experimental error. While there are no classic hydrogen bonds found in the crystal, there are weak C—H···O intra- and intermolecular interactions.

### Experimental

To a 250 ml round flask fitted with a condenser was added dicyclohexyl amine (0.1 mol), dichloromethane (15 ml) and triethylamine (0.5 ml. 4-Nitrobenzoyl chloride (0.1 mol) was added. The reaction mixture was stirred at room temperature for 1 h and then refluxed for 2 h. The product precipitated as a colorless powder, which was washed three times with water and dichloromethane. Recrystallization from ethyl acetate produced the crystals of the title compound.

### Refinement

The crystal structure is a non-merohedral twin with the twin law in the reciprocal matrix of -1, 0, 0: 0, -1, 0: 0, -1, 1 and the twin component ratio of 0.048 (1)/0.952 (1)/0.048. In the refinement the HKLF 4 reflection file format in *SHELXL* was used.

All H atoms were placed in calculated positions and then refined using the riding model approximation with atom-H lengths of 0.95 Å (CH) or 0.99 Å (CH_2_). Isotropic displacement parameters for these atoms were set to 1.2 (CH or CH_2_) times *U*_eq_ of the parent atom.

### Crystal data

**Table:**

C_19_H_26_N_2_O_3_	*Z* = 4
*M**_r_* = 330.42	*F*(000) = 712
Triclinic, *P*1	*D*_x_ = 1.267 Mg m^−^^3^
*a* = 6.1874 (3) Å	Cu *K*α radiation, λ = 1.54184 Å
*b* = 10.7109 (4) Å	Cell parameters from 6557 reflections
*c* = 26.8188 (11) Å	θ = 3.4–75.4°
α = 79.128 (4)°	µ = 0.69 mm^−^^1^
β = 89.027 (4)°	*T* = 123 K
γ = 82.883 (3)°	Plate, colorless
*V* = 1731.97 (13) Å^3^	0.29 × 0.26 × 0.07 mm

### Data collection

**Table:**

Agilent Xcalibur Ruby Gemini diffractometer	6952 independent reflections
Radiation source: Enhance (Cu) X-ray Source	6374 reflections with *I* > 2σ(*I*)
Graphite monochromator	*R*_int_ = 0.028
Detector resolution: 10.5081 pixels mm^-1^	θ_max_ = 75.6°, θ_min_ = 3.4°
ω scans	*h* = −5→7
Absorption correction: analytical (Clark & Reid, 1995)	*k* = −13→11
*T*_min_ = 0.823, *T*_max_ = 0.950	*l* = −33→33
12453 measured reflections	

### Refinement

**Table:**

Refinement on *F*^2^	Primary atom site location: structure-invariant direct methods
Least-squares matrix: full	Secondary atom site location: difference Fourier map
*R*[*F*^2^ > 2σ(*F*^2^)] = 0.080	Hydrogen site location: inferred from neighbouring sites
*wR*(*F*^2^) = 0.239	H-atom parameters constrained
*S* = 1.11	*w* = 1/[σ^2^(*F*_o_^2^) + (0.1113*P*)^2^ + 3.0338*P*] where *P* = (*F*_o_^2^ + 2*F*_c_^2^)/3
6952 reflections	(Δ/σ)_max_ < 0.001
434 parameters	Δρ_max_ = 0.41 e Å^−^^3^
0 restraints	Δρ_min_ = −0.29 e Å^−^^3^

### Special details

**Table:**

Geometry. All e.s.d.'s (except the e.s.d. in the dihedral angle between two l.s. planes) are estimated using the full covariance matrix. The cell e.s.d.'s are taken into account individually in the estimation of e.s.d.'s in distances, angles and torsion angles; correlations between e.s.d.'s in cell parameters are only used when they are defined by crystal symmetry. An approximate (isotropic) treatment of cell e.s.d.'s is used for estimating e.s.d.'s involving l.s. planes.
Refinement. Refinement of *F*^2^ against ALL reflections. The weighted *R*-factor *wR* and goodness of fit *S* are based on *F*^2^, conventional *R*-factors *R* are based on *F*, with *F* set to zero for negative *F*^2^. The threshold expression of *F*^2^ > σ(*F*^2^) is used only for calculating *R*-factors(gt) *etc*. and is not relevant to the choice of reflections for refinement. *R*-factors based on *F*^2^ are statistically about twice as large as those based on *F*, and *R*- factors based on ALL data will be even larger.

### Fractional atomic coordinates and isotropic or equivalent isotropic displacement parameters (Å^2^)

**Table:**

	*x*	*y*	*z*	*U*_iso_*/*U*_eq_	
O1A	1.2037 (5)	0.5044 (3)	1.08793 (12)	0.0488 (7)	
O2A	0.9117 (5)	0.4171 (2)	1.08150 (10)	0.0412 (6)	
O3A	0.6696 (4)	1.0644 (2)	0.95462 (9)	0.0300 (5)	
O1B	0.3898 (5)	−0.0077 (3)	0.41690 (10)	0.0426 (6)	
O2B	0.6849 (5)	0.0824 (3)	0.40932 (11)	0.0488 (7)	
O3B	0.1784 (4)	0.5172 (2)	0.54297 (9)	0.0295 (5)	
N1A	1.0181 (5)	0.5061 (3)	1.07197 (11)	0.0324 (6)	
N2A	0.5886 (4)	0.9656 (2)	0.89045 (10)	0.0241 (5)	
N1B	0.5025 (5)	0.0694 (3)	0.42687 (10)	0.0322 (6)	
N2B	0.1038 (4)	0.3626 (2)	0.60959 (10)	0.0235 (5)	
C1A	0.9244 (5)	0.6242 (3)	1.03726 (11)	0.0268 (6)	
C2A	0.7117 (6)	0.6306 (3)	1.01945 (12)	0.0289 (7)	
H2AA	0.6273	0.5621	1.0300	0.035*	
C3A	0.6276 (5)	0.7404 (3)	0.98588 (12)	0.0276 (6)	
H3AA	0.4837	0.7472	0.9729	0.033*	
C4A	0.7519 (5)	0.8398 (3)	0.97115 (11)	0.0241 (6)	
C5A	0.9626 (5)	0.8314 (3)	0.99050 (12)	0.0257 (6)	
H5AA	1.0470	0.9001	0.9806	0.031*	
C6A	1.0485 (5)	0.7220 (3)	1.02437 (12)	0.0283 (7)	
H6AA	1.1907	0.7157	1.0382	0.034*	
C7A	0.6645 (5)	0.9660 (3)	0.93753 (12)	0.0246 (6)	
C8A	0.5043 (5)	1.0884 (3)	0.85731 (11)	0.0246 (6)	
H8AA	0.4601	1.0661	0.8247	0.030*	
C9A	0.2981 (5)	1.1545 (3)	0.87833 (12)	0.0283 (7)	
H9AA	0.3326	1.1802	0.9106	0.034*	
H9AB	0.1871	1.0945	0.8853	0.034*	
C10A	0.2085 (6)	1.2733 (3)	0.83935 (14)	0.0327 (7)	
H10A	0.1599	1.2460	0.8085	0.039*	
H10B	0.0804	1.3188	0.8538	0.039*	
C11A	0.3790 (7)	1.3642 (3)	0.82482 (16)	0.0416 (9)	
H11A	0.4159	1.3989	0.8549	0.050*	
H11B	0.3183	1.4370	0.7984	0.050*	
C12A	0.5854 (6)	1.2968 (3)	0.80490 (15)	0.0371 (8)	
H12A	0.6962	1.3569	0.7978	0.045*	
H12B	0.5519	1.2704	0.7727	0.045*	
C13A	0.6769 (5)	1.1789 (3)	0.84358 (13)	0.0306 (7)	
H13A	0.8054	1.1338	0.8291	0.037*	
H13B	0.7243	1.2059	0.8746	0.037*	
C14A	0.6076 (5)	0.8494 (3)	0.86733 (11)	0.0222 (6)	
H14A	0.6707	0.7754	0.8936	0.027*	
C15A	0.7626 (5)	0.8599 (3)	0.82207 (11)	0.0251 (6)	
H15A	0.9081	0.8736	0.8332	0.030*	
H15B	0.7074	0.9344	0.7957	0.030*	
C16A	0.7815 (5)	0.7375 (3)	0.79970 (12)	0.0278 (6)	
H16A	0.8781	0.7467	0.7698	0.033*	
H16B	0.8473	0.6641	0.8252	0.033*	
C17A	0.5575 (6)	0.7111 (3)	0.78393 (13)	0.0307 (7)	
H17A	0.4961	0.7815	0.7566	0.037*	
H17B	0.5725	0.6304	0.7706	0.037*	
C18A	0.4038 (5)	0.7004 (3)	0.82904 (12)	0.0281 (6)	
H18A	0.4591	0.6252	0.8551	0.034*	
H18B	0.2583	0.6867	0.8178	0.034*	
C19A	0.3824 (5)	0.8210 (3)	0.85253 (12)	0.0256 (6)	
H19A	0.2894	0.8088	0.8830	0.031*	
H19B	0.3118	0.8948	0.8278	0.031*	
C1B	0.4191 (6)	0.1522 (3)	0.46324 (11)	0.0277 (7)	
C2B	0.2093 (6)	0.1422 (3)	0.48212 (12)	0.0279 (6)	
H2BA	0.1217	0.0842	0.4721	0.033*	
C3B	0.1331 (5)	0.2205 (3)	0.51624 (11)	0.0266 (6)	
H3BA	−0.0081	0.2151	0.5304	0.032*	
C4B	0.2611 (5)	0.3060 (3)	0.52976 (11)	0.0234 (6)	
C5B	0.4682 (5)	0.3148 (3)	0.50942 (11)	0.0261 (6)	
H5BA	0.5549	0.3742	0.5186	0.031*	
C6B	0.5487 (5)	0.2367 (3)	0.47565 (12)	0.0279 (6)	
H6BA	0.6900	0.2418	0.4616	0.033*	
C7B	0.1765 (5)	0.4039 (3)	0.56197 (11)	0.0241 (6)	
C8B	0.0134 (5)	0.4579 (3)	0.64028 (11)	0.0246 (6)	
H8BA	−0.0280	0.4079	0.6737	0.030*	
C9B	0.1805 (5)	0.5419 (3)	0.65189 (13)	0.0289 (7)	
H9BA	0.2298	0.5919	0.6198	0.035*	
H9BB	0.3088	0.4870	0.6690	0.035*	
C10B	0.0831 (6)	0.6335 (3)	0.68602 (13)	0.0319 (7)	
H10C	0.1907	0.6911	0.6912	0.038*	
H10D	0.0489	0.5838	0.7196	0.038*	
C11B	−0.1255 (6)	0.7136 (3)	0.66192 (13)	0.0300 (7)	
H11C	−0.1895	0.7700	0.6850	0.036*	
H11D	−0.0891	0.7685	0.6296	0.036*	
C12B	−0.2904 (5)	0.6276 (3)	0.65174 (12)	0.0288 (7)	
H12C	−0.4223	0.6811	0.6358	0.035*	
H12D	−0.3337	0.5768	0.6843	0.035*	
C13B	−0.1973 (5)	0.5371 (3)	0.61686 (12)	0.0262 (6)	
H13C	−0.1660	0.5872	0.5831	0.031*	
H13D	−0.3051	0.4791	0.6122	0.031*	
C14B	0.1299 (5)	0.2268 (3)	0.63518 (11)	0.0232 (6)	
H14B	0.1956	0.1748	0.6102	0.028*	
C15B	−0.0898 (5)	0.1806 (3)	0.65121 (12)	0.0262 (6)	
H15C	−0.1845	0.1916	0.6209	0.031*	
H15D	−0.1624	0.2333	0.6747	0.031*	
C16B	−0.0599 (5)	0.0395 (3)	0.67733 (13)	0.0290 (7)	
H16C	−0.2029	0.0133	0.6891	0.035*	
H16D	−0.0016	−0.0140	0.6527	0.035*	
C17B	0.0960 (6)	0.0174 (3)	0.72263 (13)	0.0322 (7)	
H17C	0.0320	0.0652	0.7486	0.039*	
H17D	0.1173	−0.0748	0.7380	0.039*	
C18B	0.3159 (5)	0.0618 (3)	0.70600 (12)	0.0291 (7)	
H18C	0.3846	0.0099	0.6818	0.035*	
H18E	0.4133	0.0488	0.7359	0.035*	
C19B	0.2872 (5)	0.2034 (3)	0.68080 (12)	0.0260 (6)	
H19E	0.4303	0.2298	0.6692	0.031*	
H19C	0.2291	0.2560	0.7058	0.031*	

### Atomic displacement parameters (Å^2^)

**Table:**

	*U*^11^	*U*^22^	*U*^33^	*U*^12^	*U*^13^	*U*^23^
O1A	0.0452 (16)	0.0435 (15)	0.0500 (17)	0.0052 (12)	−0.0178 (13)	0.0058 (12)
O2A	0.0485 (15)	0.0328 (13)	0.0373 (14)	−0.0007 (11)	0.0025 (11)	0.0032 (10)
O3A	0.0343 (12)	0.0277 (11)	0.0284 (11)	0.0000 (9)	−0.0083 (9)	−0.0083 (9)
O1B	0.0547 (16)	0.0393 (14)	0.0358 (14)	0.0027 (12)	−0.0033 (12)	−0.0174 (11)
O2B	0.0531 (17)	0.0490 (16)	0.0445 (16)	0.0040 (13)	0.0191 (13)	−0.0179 (13)
O3B	0.0346 (12)	0.0263 (11)	0.0269 (11)	−0.0022 (9)	0.0054 (9)	−0.0049 (9)
N1A	0.0390 (16)	0.0302 (15)	0.0248 (13)	0.0051 (12)	0.0004 (11)	−0.0034 (11)
N2A	0.0270 (13)	0.0229 (12)	0.0219 (12)	−0.0010 (10)	−0.0023 (10)	−0.0042 (10)
N1B	0.0428 (16)	0.0280 (14)	0.0233 (13)	0.0077 (12)	−0.0006 (12)	−0.0061 (11)
N2B	0.0251 (12)	0.0230 (12)	0.0220 (12)	0.0004 (10)	0.0014 (10)	−0.0053 (10)
C1A	0.0324 (16)	0.0271 (15)	0.0190 (14)	0.0055 (12)	−0.0010 (12)	−0.0050 (11)
C2A	0.0326 (17)	0.0288 (16)	0.0253 (15)	−0.0046 (13)	0.0028 (12)	−0.0049 (12)
C3A	0.0261 (15)	0.0323 (16)	0.0238 (15)	0.0005 (12)	−0.0016 (12)	−0.0060 (12)
C4A	0.0247 (14)	0.0273 (15)	0.0200 (14)	0.0021 (12)	−0.0016 (11)	−0.0073 (11)
C5A	0.0255 (15)	0.0276 (15)	0.0239 (14)	−0.0005 (12)	−0.0024 (11)	−0.0063 (12)
C6A	0.0266 (15)	0.0331 (17)	0.0249 (15)	0.0032 (13)	−0.0037 (12)	−0.0087 (12)
C7A	0.0217 (14)	0.0267 (15)	0.0249 (15)	−0.0018 (11)	−0.0014 (11)	−0.0043 (12)
C8A	0.0258 (15)	0.0236 (14)	0.0233 (14)	0.0006 (12)	−0.0038 (11)	−0.0035 (11)
C9A	0.0272 (15)	0.0273 (15)	0.0290 (16)	0.0034 (12)	−0.0037 (12)	−0.0056 (12)
C10A	0.0314 (17)	0.0275 (16)	0.0373 (18)	0.0051 (13)	−0.0084 (13)	−0.0060 (13)
C11A	0.046 (2)	0.0243 (16)	0.051 (2)	0.0019 (15)	−0.0161 (17)	−0.0024 (15)
C12A	0.0381 (19)	0.0306 (17)	0.0395 (19)	−0.0083 (14)	−0.0074 (15)	0.0051 (14)
C13A	0.0296 (16)	0.0267 (16)	0.0332 (17)	−0.0019 (13)	−0.0057 (13)	−0.0006 (13)
C14A	0.0230 (14)	0.0221 (14)	0.0210 (14)	0.0001 (11)	−0.0025 (11)	−0.0050 (11)
C15A	0.0225 (14)	0.0290 (15)	0.0235 (14)	−0.0016 (12)	−0.0015 (11)	−0.0051 (12)
C16A	0.0281 (15)	0.0303 (16)	0.0239 (15)	0.0021 (12)	0.0012 (12)	−0.0067 (12)
C17A	0.0342 (17)	0.0314 (16)	0.0275 (16)	−0.0003 (13)	−0.0022 (13)	−0.0097 (13)
C18A	0.0283 (15)	0.0278 (15)	0.0291 (16)	−0.0044 (12)	−0.0022 (12)	−0.0072 (12)
C19A	0.0238 (14)	0.0275 (15)	0.0253 (14)	−0.0021 (12)	−0.0002 (11)	−0.0055 (12)
C1B	0.0355 (17)	0.0264 (15)	0.0182 (14)	0.0064 (13)	0.0006 (12)	−0.0032 (11)
C2B	0.0342 (16)	0.0257 (15)	0.0237 (15)	−0.0023 (12)	−0.0044 (12)	−0.0049 (12)
C3B	0.0270 (15)	0.0298 (15)	0.0218 (14)	−0.0003 (12)	0.0012 (11)	−0.0037 (12)
C4B	0.0274 (15)	0.0232 (14)	0.0175 (13)	0.0019 (11)	−0.0009 (11)	−0.0015 (11)
C5B	0.0274 (15)	0.0279 (15)	0.0217 (14)	−0.0010 (12)	−0.0014 (11)	−0.0027 (11)
C6B	0.0281 (15)	0.0298 (16)	0.0229 (14)	0.0021 (12)	0.0031 (12)	−0.0015 (12)
C7B	0.0244 (14)	0.0256 (15)	0.0224 (14)	−0.0023 (11)	−0.0002 (11)	−0.0055 (11)
C8B	0.0279 (15)	0.0258 (15)	0.0202 (13)	−0.0003 (12)	0.0027 (11)	−0.0066 (11)
C9B	0.0281 (16)	0.0300 (16)	0.0294 (16)	−0.0013 (13)	0.0001 (12)	−0.0092 (13)
C10B	0.0345 (17)	0.0339 (17)	0.0299 (16)	−0.0054 (14)	−0.0018 (13)	−0.0118 (13)
C11B	0.0356 (17)	0.0264 (15)	0.0279 (16)	0.0004 (13)	0.0012 (13)	−0.0081 (12)
C12B	0.0298 (16)	0.0279 (15)	0.0283 (15)	0.0003 (12)	0.0037 (12)	−0.0072 (12)
C13B	0.0261 (15)	0.0269 (15)	0.0254 (15)	0.0003 (12)	−0.0010 (11)	−0.0072 (12)
C14B	0.0262 (14)	0.0231 (14)	0.0195 (13)	0.0001 (11)	0.0004 (11)	−0.0042 (11)
C15B	0.0217 (14)	0.0268 (15)	0.0298 (15)	−0.0011 (12)	−0.0028 (12)	−0.0052 (12)
C16B	0.0267 (15)	0.0259 (15)	0.0346 (17)	−0.0056 (12)	0.0041 (13)	−0.0056 (13)
C17B	0.0338 (17)	0.0298 (16)	0.0301 (16)	−0.0013 (13)	0.0054 (13)	0.0000 (13)
C18B	0.0281 (16)	0.0316 (16)	0.0238 (15)	0.0034 (13)	−0.0025 (12)	0.0000 (12)
C19B	0.0214 (14)	0.0288 (15)	0.0264 (15)	0.0000 (11)	−0.0022 (11)	−0.0034 (12)

### Geometric parameters (Å, º)

**Table:**

O1A—N1A	1.229 (4)	C17A—H17A	0.9900
O2A—N1A	1.211 (4)	C17A—H17B	0.9900
O3A—C7A	1.230 (4)	C18A—C19A	1.532 (4)
O1B—N1B	1.213 (4)	C18A—H18A	0.9900
O2B—N1B	1.229 (4)	C18A—H18B	0.9900
O3B—C7B	1.226 (4)	C19A—H19A	0.9900
N1A—C1A	1.481 (4)	C19A—H19B	0.9900
N2A—C7A	1.356 (4)	C1B—C6B	1.369 (5)
N2A—C8A	1.483 (4)	C1B—C2B	1.395 (5)
N2A—C14A	1.483 (4)	C2B—C3B	1.393 (4)
N1B—C1B	1.483 (4)	C2B—H2BA	0.9500
N2B—C7B	1.359 (4)	C3B—C4B	1.383 (5)
N2B—C14B	1.477 (4)	C3B—H3BA	0.9500
N2B—C8B	1.480 (4)	C4B—C5B	1.391 (4)
C1A—C6A	1.364 (5)	C4B—C7B	1.520 (4)
C1A—C2A	1.397 (5)	C5B—C6B	1.391 (4)
C2A—C3A	1.389 (5)	C5B—H5BA	0.9500
C2A—H2AA	0.9500	C6B—H6BA	0.9500
C3A—C4A	1.384 (5)	C8B—C9B	1.524 (4)
C3A—H3AA	0.9500	C8B—C13B	1.538 (4)
C4A—C5A	1.398 (4)	C8B—H8BA	1.0000
C4A—C7A	1.519 (4)	C9B—C10B	1.528 (4)
C5A—C6A	1.393 (4)	C9B—H9BA	0.9900
C5A—H5AA	0.9500	C9B—H9BB	0.9900
C6A—H6AA	0.9500	C10B—C11B	1.538 (5)
C8A—C13A	1.524 (5)	C10B—H10C	0.9900
C8A—C9A	1.533 (4)	C10B—H10D	0.9900
C8A—H8AA	1.0000	C11B—C12B	1.517 (5)
C9A—C10A	1.537 (4)	C11B—H11C	0.9900
C9A—H9AA	0.9900	C11B—H11D	0.9900
C9A—H9AB	0.9900	C12B—C13B	1.527 (4)
C10A—C11A	1.520 (5)	C12B—H12C	0.9900
C10A—H10A	0.9900	C12B—H12D	0.9900
C10A—H10B	0.9900	C13B—H13C	0.9900
C11A—C12A	1.527 (6)	C13B—H13D	0.9900
C11A—H11A	0.9900	C14B—C15B	1.530 (4)
C11A—H11B	0.9900	C14B—C19B	1.540 (4)
C12A—C13A	1.530 (4)	C14B—H14B	1.0000
C12A—H12A	0.9900	C15B—C16B	1.531 (4)
C12A—H12B	0.9900	C15B—H15C	0.9900
C13A—H13A	0.9900	C15B—H15D	0.9900
C13A—H13B	0.9900	C16B—C17B	1.529 (5)
C14A—C15A	1.530 (4)	C16B—H16C	0.9900
C14A—C19A	1.536 (4)	C16B—H16D	0.9900
C14A—H14A	1.0000	C17B—C18B	1.528 (5)
C15A—C16A	1.533 (4)	C17B—H17C	0.9900
C15A—H15A	0.9900	C17B—H17D	0.9900
C15A—H15B	0.9900	C18B—C19B	1.530 (4)
C16A—C17A	1.530 (5)	C18B—H18C	0.9900
C16A—H16A	0.9900	C18B—H18E	0.9900
C16A—H16B	0.9900	C19B—H19E	0.9900
C17A—C18A	1.522 (5)	C19B—H19C	0.9900
			
O2A—N1A—O1A	124.4 (3)	C18A—C19A—C14A	110.5 (2)
O2A—N1A—C1A	118.6 (3)	C18A—C19A—H19A	109.6
O1A—N1A—C1A	116.9 (3)	C14A—C19A—H19A	109.6
C7A—N2A—C8A	119.5 (2)	C18A—C19A—H19B	109.6
C7A—N2A—C14A	123.4 (3)	C14A—C19A—H19B	109.6
C8A—N2A—C14A	116.7 (2)	H19A—C19A—H19B	108.1
O1B—N1B—O2B	124.1 (3)	C6B—C1B—C2B	123.2 (3)
O1B—N1B—C1B	118.7 (3)	C6B—C1B—N1B	118.7 (3)
O2B—N1B—C1B	117.2 (3)	C2B—C1B—N1B	118.0 (3)
C7B—N2B—C14B	123.6 (2)	C3B—C2B—C1B	117.4 (3)
C7B—N2B—C8B	119.3 (2)	C3B—C2B—H2BA	121.3
C14B—N2B—C8B	116.8 (2)	C1B—C2B—H2BA	121.3
C6A—C1A—C2A	122.9 (3)	C4B—C3B—C2B	120.6 (3)
C6A—C1A—N1A	118.7 (3)	C4B—C3B—H3BA	119.7
C2A—C1A—N1A	118.4 (3)	C2B—C3B—H3BA	119.7
C3A—C2A—C1A	117.8 (3)	C3B—C4B—C5B	120.3 (3)
C3A—C2A—H2AA	121.1	C3B—C4B—C7B	122.6 (3)
C1A—C2A—H2AA	121.1	C5B—C4B—C7B	116.8 (3)
C4A—C3A—C2A	120.5 (3)	C4B—C5B—C6B	120.2 (3)
C4A—C3A—H3AA	119.8	C4B—C5B—H5BA	119.9
C2A—C3A—H3AA	119.8	C6B—C5B—H5BA	119.9
C3A—C4A—C5A	120.2 (3)	C1B—C6B—C5B	118.3 (3)
C3A—C4A—C7A	123.1 (3)	C1B—C6B—H6BA	120.9
C5A—C4A—C7A	116.5 (3)	C5B—C6B—H6BA	120.9
C6A—C5A—C4A	119.9 (3)	O3B—C7B—N2B	123.6 (3)
C6A—C5A—H5AA	120.1	O3B—C7B—C4B	117.2 (3)
C4A—C5A—H5AA	120.1	N2B—C7B—C4B	119.2 (3)
C1A—C6A—C5A	118.6 (3)	N2B—C8B—C9B	112.9 (3)
C1A—C6A—H6AA	120.7	N2B—C8B—C13B	112.0 (2)
C5A—C6A—H6AA	120.7	C9B—C8B—C13B	112.3 (3)
O3A—C7A—N2A	123.3 (3)	N2B—C8B—H8BA	106.4
O3A—C7A—C4A	117.5 (3)	C9B—C8B—H8BA	106.4
N2A—C7A—C4A	119.2 (3)	C13B—C8B—H8BA	106.4
N2A—C8A—C13A	113.2 (2)	C8B—C9B—C10B	110.8 (3)
N2A—C8A—C9A	112.7 (3)	C8B—C9B—H9BA	109.5
C13A—C8A—C9A	111.9 (3)	C10B—C9B—H9BA	109.5
N2A—C8A—H8AA	106.1	C8B—C9B—H9BB	109.5
C13A—C8A—H8AA	106.1	C10B—C9B—H9BB	109.5
C9A—C8A—H8AA	106.1	H9BA—C9B—H9BB	108.1
C8A—C9A—C10A	109.4 (3)	C9B—C10B—C11B	110.5 (3)
C8A—C9A—H9AA	109.8	C9B—C10B—H10C	109.6
C10A—C9A—H9AA	109.8	C11B—C10B—H10C	109.6
C8A—C9A—H9AB	109.8	C9B—C10B—H10D	109.6
C10A—C9A—H9AB	109.8	C11B—C10B—H10D	109.6
H9AA—C9A—H9AB	108.2	H10C—C10B—H10D	108.1
C11A—C10A—C9A	111.6 (3)	C12B—C11B—C10B	110.8 (3)
C11A—C10A—H10A	109.3	C12B—C11B—H11C	109.5
C9A—C10A—H10A	109.3	C10B—C11B—H11C	109.5
C11A—C10A—H10B	109.3	C12B—C11B—H11D	109.5
C9A—C10A—H10B	109.3	C10B—C11B—H11D	109.5
H10A—C10A—H10B	108.0	H11C—C11B—H11D	108.1
C10A—C11A—C12A	111.5 (3)	C11B—C12B—C13B	111.4 (3)
C10A—C11A—H11A	109.3	C11B—C12B—H12C	109.4
C12A—C11A—H11A	109.3	C13B—C12B—H12C	109.4
C10A—C11A—H11B	109.3	C11B—C12B—H12D	109.4
C12A—C11A—H11B	109.3	C13B—C12B—H12D	109.4
H11A—C11A—H11B	108.0	H12C—C12B—H12D	108.0
C11A—C12A—C13A	110.9 (3)	C12B—C13B—C8B	109.6 (3)
C11A—C12A—H12A	109.5	C12B—C13B—H13C	109.7
C13A—C12A—H12A	109.5	C8B—C13B—H13C	109.7
C11A—C12A—H12B	109.5	C12B—C13B—H13D	109.7
C13A—C12A—H12B	109.5	C8B—C13B—H13D	109.7
H12A—C12A—H12B	108.0	H13C—C13B—H13D	108.2
C8A—C13A—C12A	110.2 (3)	N2B—C14B—C15B	111.6 (2)
C8A—C13A—H13A	109.6	N2B—C14B—C19B	111.3 (2)
C12A—C13A—H13A	109.6	C15B—C14B—C19B	111.0 (2)
C8A—C13A—H13B	109.6	N2B—C14B—H14B	107.6
C12A—C13A—H13B	109.6	C15B—C14B—H14B	107.6
H13A—C13A—H13B	108.1	C19B—C14B—H14B	107.6
N2A—C14A—C15A	111.7 (2)	C14B—C15B—C16B	110.9 (2)
N2A—C14A—C19A	110.9 (2)	C14B—C15B—H15C	109.5
C15A—C14A—C19A	111.5 (2)	C16B—C15B—H15C	109.5
N2A—C14A—H14A	107.5	C14B—C15B—H15D	109.5
C15A—C14A—H14A	107.5	C16B—C15B—H15D	109.5
C19A—C14A—H14A	107.5	H15C—C15B—H15D	108.0
C14A—C15A—C16A	110.3 (3)	C17B—C16B—C15B	111.0 (3)
C14A—C15A—H15A	109.6	C17B—C16B—H16C	109.4
C16A—C15A—H15A	109.6	C15B—C16B—H16C	109.4
C14A—C15A—H15B	109.6	C17B—C16B—H16D	109.4
C16A—C15A—H15B	109.6	C15B—C16B—H16D	109.4
H15A—C15A—H15B	108.1	H16C—C16B—H16D	108.0
C17A—C16A—C15A	110.8 (3)	C18B—C17B—C16B	110.7 (3)
C17A—C16A—H16A	109.5	C18B—C17B—H17C	109.5
C15A—C16A—H16A	109.5	C16B—C17B—H17C	109.5
C17A—C16A—H16B	109.5	C18B—C17B—H17D	109.5
C15A—C16A—H16B	109.5	C16B—C17B—H17D	109.5
H16A—C16A—H16B	108.1	H17C—C17B—H17D	108.1
C18A—C17A—C16A	110.5 (3)	C17B—C18B—C19B	110.6 (3)
C18A—C17A—H17A	109.6	C17B—C18B—H18C	109.5
C16A—C17A—H17A	109.6	C19B—C18B—H18C	109.5
C18A—C17A—H17B	109.6	C17B—C18B—H18E	109.5
C16A—C17A—H17B	109.6	C19B—C18B—H18E	109.5
H17A—C17A—H17B	108.1	H18C—C18B—H18E	108.1
C17A—C18A—C19A	111.6 (3)	C18B—C19B—C14B	110.4 (3)
C17A—C18A—H18A	109.3	C18B—C19B—H19E	109.6
C19A—C18A—H18A	109.3	C14B—C19B—H19E	109.6
C17A—C18A—H18B	109.3	C18B—C19B—H19C	109.6
C19A—C18A—H18B	109.3	C14B—C19B—H19C	109.6
H18A—C18A—H18B	108.0	H19E—C19B—H19C	108.1
			
O2A—N1A—C1A—C6A	−176.6 (3)	O1B—N1B—C1B—C6B	178.1 (3)
O1A—N1A—C1A—C6A	1.2 (4)	O2B—N1B—C1B—C6B	−0.7 (4)
O2A—N1A—C1A—C2A	3.9 (4)	O1B—N1B—C1B—C2B	−3.5 (4)
O1A—N1A—C1A—C2A	−178.4 (3)	O2B—N1B—C1B—C2B	177.7 (3)
C6A—C1A—C2A—C3A	2.1 (5)	C6B—C1B—C2B—C3B	−1.7 (5)
N1A—C1A—C2A—C3A	−178.3 (3)	N1B—C1B—C2B—C3B	180.0 (3)
C1A—C2A—C3A—C4A	−0.4 (5)	C1B—C2B—C3B—C4B	1.2 (5)
C2A—C3A—C4A—C5A	−0.9 (5)	C2B—C3B—C4B—C5B	−0.1 (5)
C2A—C3A—C4A—C7A	−175.6 (3)	C2B—C3B—C4B—C7B	172.8 (3)
C3A—C4A—C5A—C6A	0.6 (4)	C3B—C4B—C5B—C6B	−0.6 (4)
C7A—C4A—C5A—C6A	175.7 (3)	C7B—C4B—C5B—C6B	−173.9 (3)
C2A—C1A—C6A—C5A	−2.4 (5)	C2B—C1B—C6B—C5B	1.1 (5)
N1A—C1A—C6A—C5A	178.1 (3)	N1B—C1B—C6B—C5B	179.4 (3)
C4A—C5A—C6A—C1A	1.0 (5)	C4B—C5B—C6B—C1B	0.1 (5)
C8A—N2A—C7A—O3A	−0.7 (5)	C14B—N2B—C7B—O3B	−171.0 (3)
C14A—N2A—C7A—O3A	172.1 (3)	C8B—N2B—C7B—O3B	2.4 (5)
C8A—N2A—C7A—C4A	−179.7 (3)	C14B—N2B—C7B—C4B	9.0 (4)
C14A—N2A—C7A—C4A	−6.9 (4)	C8B—N2B—C7B—C4B	−177.7 (3)
C3A—C4A—C7A—O3A	121.5 (3)	C3B—C4B—C7B—O3B	−119.6 (3)
C5A—C4A—C7A—O3A	−53.4 (4)	C5B—C4B—C7B—O3B	53.5 (4)
C3A—C4A—C7A—N2A	−59.5 (4)	C3B—C4B—C7B—N2B	60.4 (4)
C5A—C4A—C7A—N2A	125.6 (3)	C5B—C4B—C7B—N2B	−126.5 (3)
C7A—N2A—C8A—C13A	63.0 (4)	C7B—N2B—C8B—C9B	−63.0 (4)
C14A—N2A—C8A—C13A	−110.2 (3)	C14B—N2B—C8B—C9B	110.8 (3)
C7A—N2A—C8A—C9A	−65.2 (4)	C7B—N2B—C8B—C13B	64.9 (4)
C14A—N2A—C8A—C9A	121.5 (3)	C14B—N2B—C8B—C13B	−121.2 (3)
N2A—C8A—C9A—C10A	−174.3 (3)	N2B—C8B—C9B—C10B	−176.9 (3)
C13A—C8A—C9A—C10A	56.8 (3)	C13B—C8B—C9B—C10B	55.3 (3)
C8A—C9A—C10A—C11A	−55.6 (4)	C8B—C9B—C10B—C11B	−55.3 (4)
C9A—C10A—C11A—C12A	55.9 (4)	C9B—C10B—C11B—C12B	57.1 (4)
C10A—C11A—C12A—C13A	−55.7 (4)	C10B—C11B—C12B—C13B	−58.2 (4)
N2A—C8A—C13A—C12A	173.9 (3)	C11B—C12B—C13B—C8B	56.5 (3)
C9A—C8A—C13A—C12A	−57.4 (4)	N2B—C8B—C13B—C12B	176.5 (3)
C11A—C12A—C13A—C8A	56.0 (4)	C9B—C8B—C13B—C12B	−55.3 (3)
C7A—N2A—C14A—C15A	−112.5 (3)	C7B—N2B—C14B—C15B	−122.7 (3)
C8A—N2A—C14A—C15A	60.4 (3)	C8B—N2B—C14B—C15B	63.7 (3)
C7A—N2A—C14A—C19A	122.5 (3)	C7B—N2B—C14B—C19B	112.6 (3)
C8A—N2A—C14A—C19A	−64.6 (3)	C8B—N2B—C14B—C19B	−60.9 (3)
N2A—C14A—C15A—C16A	179.0 (2)	N2B—C14B—C15B—C16B	179.6 (2)
C19A—C14A—C15A—C16A	−56.3 (3)	C19B—C14B—C15B—C16B	−55.6 (3)
C14A—C15A—C16A—C17A	57.2 (3)	C14B—C15B—C16B—C17B	55.9 (3)
C15A—C16A—C17A—C18A	−57.5 (4)	C15B—C16B—C17B—C18B	−57.0 (4)
C16A—C17A—C18A—C19A	56.8 (4)	C16B—C17B—C18B—C19B	57.8 (4)
C17A—C18A—C19A—C14A	−55.5 (3)	C17B—C18B—C19B—C14B	−57.2 (3)
N2A—C14A—C19A—C18A	−179.6 (2)	N2B—C14B—C19B—C18B	−178.7 (2)
C15A—C14A—C19A—C18A	55.3 (3)	C15B—C14B—C19B—C18B	56.3 (3)

### Hydrogen-bond geometry (Å, º)

**Table:**

*D*—H···*A*	*D*—H	H···*A*	*D*···*A*	*D*—H···*A*
C5*A*—H5*AA*···O3*A*^i^	0.95	2.60	3.155 (4)	118
C5*B*—H5*BA*···O3*B*^ii^	0.95	2.54	3.148 (4)	122
C9*A*—H9*AA*···O3*A*	0.99	2.48	3.044 (4)	115
C9*B*—H9*BA*···O3*B*	0.99	2.38	2.983 (4)	118
C13*A*—H13*B*···O3*A*	0.99	2.43	3.001 (4)	116
C13*B*—H13*C*···O3*B*	0.99	2.47	3.043 (4)	117

Symmetry codes: (i) −*x*+2, −*y*+2, −*z*+2; (ii) −*x*+1, −*y*+1, −*z*+1.
